# Repressor logic modules assembled by rolling circle amplification platform to construct a set of logic gates

**DOI:** 10.1038/srep37477

**Published:** 2016-11-21

**Authors:** Hua Wei, Bo Hu, Suming Tang, Guojie Zhao, Yifu Guan

**Affiliations:** 1 Animal Science and Veterinary Medicine College, Shenyang Agricultural University, #120 Dongling Road, Shenyang, Liaoning, 110866, China; 2 Department of Biochemistry and Molecular Biology, China Medical University, #77 Puhe Road, Shenyang, Liaoning, 110122, China

## Abstract

Small molecule metabolites and their allosterically regulated repressors play an important role in many gene expression and metabolic disorder processes. These natural sensors, though valuable as good logic switches, have rarely been employed without transcription machinery in cells. Here, two pairs of repressors, which function in opposite ways, were cloned, purified and used to control DNA replication in rolling circle amplification (RCA) *in vitro*. By using metabolites and repressors as inputs, RCA signals as outputs, four basic logic modules were constructed successfully. To achieve various logic computations based on these basic modules, we designed series and parallel strategies of circular templates, which can further assemble these repressor modules in an RCA platform to realize twelve two-input Boolean logic gates and a three-input logic gate. The RCA-output and RCA-assembled platform was proved to be easy and flexible for complex logic processes and might have application potential in molecular computing and synthetic biology.

One of the most significant characteristics in electric circuits is their ability to form complex logic circuits from basic logic modules. However, this ability is still a problem for biocomputing. Though molecular reaction in solution enables biocomputing system great potential of parallel process, lacking a spatial organization platform presents a great challenge for constructing modular process. DNA is actually a natural scaffold which has been used to recruit other molecules in various fields^1^. It has the potential to be exploited as a perfect macromolecule platform for modular logic process. Though tremendous progresses have been achieved for the nucleic acid-based logic machine, since it was introduced by Adleman^2^,^3^,^4^, how to construct and assemble basic logic modules in an easy way is still challenging and attracting.

Transcription factors (TFs) are one kind of DNA-binding proteins, which contact different promoters and control downstream gene transcription. They also act as environmental sensors, since various key metabolic small molecules are their allosteric regulators^5^,^6^. Upon binding these metabolites, they change their DNA-binding ability as switches, function the on-off state of the target gene expression^7^. The found, to be found, and engineered repressors in nature constitute abundant candidates for molecular computing^8^,^9^. As a kind of natural molecule switches, their ability in DNA sequence recognition, efficiency in gene regulation and diversity have attracted increasing interests in logic computation and synthetic biology^10^,^11^,^12^,^13^. However, the reported outputs of TF-controlled logic gates are mainly dependent on the transcription and expression machinery in cells. The whole process is time-consuming and laborious. The operation efficiency is affected by complex factors, such as cell growth rates, plasmid maintenance, and gene expression efficiency in a complex genome context^14^. Therefore, to transmit the downstream output in a simple way will surely facilitate their applications as computing modules.

In addition, many small molecules play a key role in metabolic process. Their assays are significant in disease diagnosis, biosample analysis, especially analyzing multiple small molecules in one system. Though some attempts on aptamers have achieved great progress in detecting some small molecules, such as ATP^15^,^16^, metal ions^17^,^18^, numerous significant metabolites face challenges to find their corresponding aptamers. Moreover, some of them are found to be closely related to diseases, such as L-tryptophan (L-Trp) in immune and mental disorders^19^, and S-adenosylmethionine (SAM) in lung cancer^20^. In these cases, metabolite-regulated allosteric TFs might provide another alternative route for metabolites assays. Consequently, an easy TF signal output way in a cell-free system might be helpful to accelerate mining and application of these natural protein switches in this field.

Rolling circle amplification (RCA) is an isothermal DNA replication way. Strand-displacing DNA polymerases extend primer round and round along a circular template to produce long tandem singe-stranded DNA. With the advantages of easy-operation, fast-reaction, label-free, and signal amplification, RCA has been widely exploited in nucleic acids detections, and recently has been found promising in miRNA assays and Point of Care Testing (POCT)^21^,^22^,^23^. Some attempts have also been made to detect small molecules and proteins by RCA depending on the principle of aptamer and antibody^24^,^25^,^26^. Moreover, RCA has also derived a variety of signal output means, such as fluorescence, colorimetric, electronic, physicochemical and nanopore analyses^27^,^28^,^29^,^30^,^31^, which are extremely desirable in logic operations. However, in the field of molecular computing, RCA was rarely employed by logic operations.

Just recently, we presented a repressor-RCA concept, and succeeded in detecting L-Trp by repressor TrpR controlled RCA^32^. Inspired by this finding, we thought that regulatory unit to control DNA replication rather than transcription might provide novel TF logic modules for biocomputing *in vitro*. According to different allosteric regulation forms, some metabolites decrease repressors’ DNA-binding ability by allosteric effects, and are called inducers. Some increase repressors’ DNA-binding ability, and are called anti-inducers^33^. Inducers and anti-inducers regulate repressors in opposite way, suggesting a very useful ‘NOT’ mode in logic computation (Fig. 1). Consequently, we deliberately selected one pair of inducer-regulated repressors (LacI and GalR) and one pair of anti-inducer-regulated repressors (TrpR and MetJ) as a model for logic process illustration. LacI and GalR belong to LacI/GalR family, and they bind their recognition sequences in the absence of their inducers (IPTG and D-Gal). After adding their inducers, LacI or GalR releases their bound DNA^34^,^35^. For TrpR and MetJ, only in the presence of their anti-inducers (L-Trp and SAM), repressors can bind their recognition sequences^36^,^37^.

We cloned, expressed and purified these repressors (TrpR, LacI, GalR and MetJ) (Figs S1~S3). Then we verified their feasibility to be allosterically regulated by corresponding metabolites (IPTG, D-Gal and SAM) to control RCA reaction. By using these two pairs of regulatory units, we successfully constructed four basic logic modules in a simple and convenient way. Moreover, we designed series and parallel strategies through a circular template to accomplish ‘AND’ and ‘OR’ computation of the basic regular modules, respectively. Based on this principle, we accomplished constructing a set of two-input and three-input logic gates. This provided a new and flexible way for multi-responsive logic circuit construction.

## Results and Discussion

### Constructing four basic logic modules

For initial logic operation, we constructed four basic logic modules. We embedded LacI recognition sequence in circular template. Without LacI and IPTG, phi29 DNA polymerase extended primer along the circular template to produce large amounts of RCA products, giving an obvious increased fluorescent signal. When LacI was added, it bound the circular template by the recognition sequence, and prohibited DNA polymerase from RCA. When IPTG was also added, it induced LacI allosterically to release the circular template; therefore, the RCA occurred again. We defined IPTG and LacI as two inputs, the RCA rate was measured and normalized as the output. An ORN logic gate was constructed. The threshold was defined as 0.5. When relative RCA rate (RRR) was above 0.5, the output was considered as 1, and when RRR was below 0.5, the output was considered as 0 (Fig. S4A). In addition, we also designed GalR recognition sequence in circular template. With GalR and D-Gal as inputs, we succeeded in constructing another ORN gate (Fig. S4B).

We also designed TrpR recognition sequence in circular template. Either we added TrpR protein or added L-Trp, the RCA process was not interfered with. Only when we added both TrpR and L-Trp, the allosteric protein could bind to the circular template, resulting in an inhibited RCA. Using L-Trp and TrpR as inputs, RCA rates as outputs, we acquired a NAND gate (Fig. S4C). In addition, we embedded MetJ recognition sequence in circular template. Only when both SAM and MetJ were added, was the RCA process reduced. Thus, we acquired another NAND gate (Fig. S4D).

These results also verified that LacI, GalR and MetJ, except TrpR, can efficiently control RCA process *in vitro*. Moreover, the whole process can be completed in 20 minutes. Therefore, the repressor-RCA was proved to be effective as a universal and convenient strategy for many metabolites and corresponding allosteric repressors.

### Series and parallel connection of basic modules

Logic combination by basic modules is a key principle in electronic circuits. It can construct complex logic relationship to set out logic computation. However, the logic combination is especially a challenge in the biomolecular computing system. Based on RCA principle, DNA polymerase runs along circular template. Thus, any site along the circular template bound by repressors can stop polymerization, and the binding one circular template of repressor cannot affect another circular template in the reaction system. Therefore, a series computation strategy can be established by designing different repressor recognition sequences in series in one template, and a parallel computation can be realized by designing different repressor recognition sequences in different circular templates. Using this combination strategy, six two-input logic gates were constructed systematically.

Different modules function in one system requires orthogonal characteristic of these modules^8^. LacI and GalR belong to LacI/GalR family, and MetJ and TrpR share some similar characteristics in size and functions^38^,^39^. The minimal cross-reactivity between inducers, anti-inducers, allosteric repressors and the recognized DNA sequences is crucial in realizing complicated logic process in one reaction system. The specific sequence needs to be precisely identified by certain repressor, and one metabolite only allosterically regulates one repressor. For each recognition sequence we used, the four repressors (LacI, GalR, MetJ and TrpR) were all tested for their specificity (Fig. S5). Though we found RCA suppression ability was relatively weaker for MetJ than for other three repressors, four repressors all had specific sequence recognition capability. Then we tested metabolites specificity for regulating repressors to control RCA. Each repressor was tested by four metabolites (IPTG, D-Gal, SAM and L-Trp), respectively (Fig. S6). Results showed that a high concentration of D-Gal can slightly induce LacI, thus we chose a 5 mM concentration D-Gal to avoid cross-reactivity (Fig. S7A). We also found a high concentration of SAM can bind TrpR to suppress RCA, so we lowered SAM concentration to 1 mM to reduce nonspecific regulation (Fig. S7B). On these concentration levels, the metabolites, repressors and their binding sequences can orthogonally interacted.

**Figure 2:**
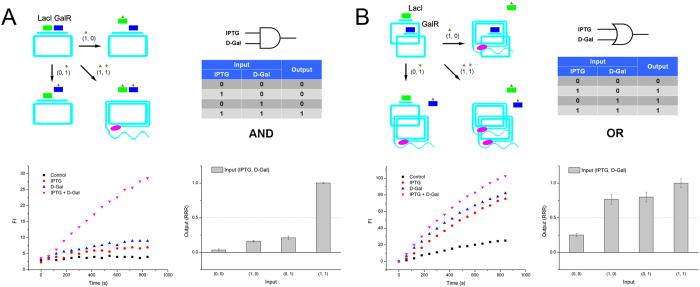
(**A**) AND gate of repressor-RCA, schematic, truth table, RCA curve and rate column. (**B**) OR gate of repressor-RCA, schematic, truth table, RCA curve and rate column. Green rectangle: LacI; blue rectangle: GalR; brown triangle: IPTG; brown diamond: D-Gal; pink oval: DNA polymerase.

After orthogonal verification, we series connected two inducer-regulated modules (LacI and GalR) in one circular template. By adding LacI and GalR protein, they both bound to the same circular template as an initial state. IPTG and D-Gal were added as inputs. When one input (IPTG or D-Gal) was added, only one repressor (LacI or GalR) was released. Thus, RCA can be still inhibited by the other bound repressor. Only if both inputs were added, the two repressors were all released, and RCA can occur. Therefore, an AND gate was constructed successfully (Fig. 2A). Then we parallel connected LacI and GalR modules by mixing their individual circular templates in the same reaction system. After adding LacI and GalR, both of the two kinds of circular templates were occupied by the two repressors. By adding either of the two inducers (IPTG or D-Gal), one kind of template was released to carry out RCA, and produced fluorescence signals. This was the characteristic of OR gate (Fig. 2B).

**Figure 3:**
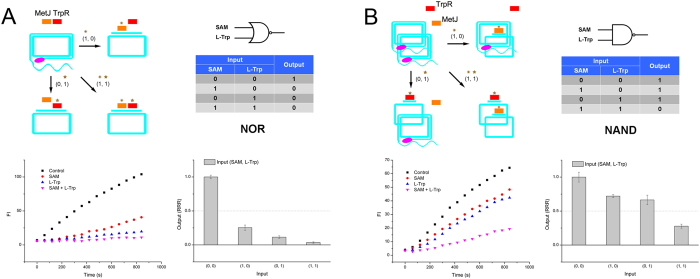
(**A**) NOR gate of repressor-RCA, schematic, truth table, RCA curve and rate column. (**B**) NAND gate of repressor-RCA, schematic, truth table, RCA curve and rate column. Orange rectangle: MetJ; red rectangle: TrpR; brown exploding star: SAM; brown star: L-Trp; pink oval: DNA polymerase.

Next, we series connected two anti-inducer-regulated modules (MetJ and TrpR) in one circular template in the presence of both MetJ and TrpR. When SAM was added, MetJ was able to bind the circular template to suppress RCA. When L-Trp was added, it regulated TrpR to bind template to inhibit RCA. When both SAM and L-Trp were added, the two repressors bound template together, and no RCA occurred. Therefore, a NOR gate was presented unambiguously (Fig. 3A). We then set out to parallel connect MetJ and TrpR modules by mixing their individual circular templates together in the presence of these two repressors. In this case, only both of the two anti-inducers (SAM and L-Trp) were presented, the two kinds of circular templates can be inhibited completely, and produced a ‘0’ output. In other cases, at least one kind of circular template can initiate the emission of RCA fluorescence signal, representing a ‘1’ output. This was the characteristic of NAND gate (Fig. 3B).

**Figure 4:**
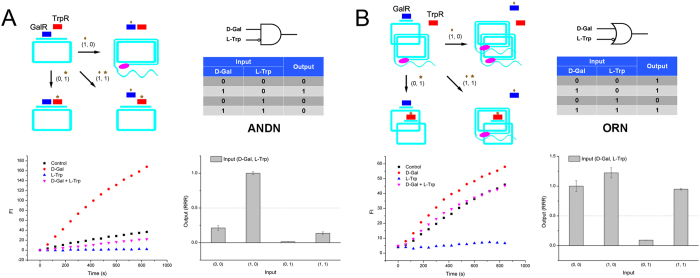
(**A**) ANDN (INH) gate of repressor-RCA, schematic, truth table, RCA curve and rate column. (**B**) ORN gate of repressor-RCA, schematic, truth table, RCA curve and rate column. Blue rectangle: GalR; red rectangle: TrpR; brown diamond: D-Gal; brown star: L-Trp; pink oval: DNA polymerase.

Moreover, we series connected an inducer-regulated module (GalR) and an anti-inducer-regulated module (TrpR) in one circular template. In the presence of GalR and TrpR, only GalR bound the circular template to inhibit RCA in an initial state. When D-Gal was added, GalR was regulated by inducer and released template to carry out RCA, therefore giving a ‘1’ output. When L-Trp was added, TrpR was triggered to bind template along with GalR, remaining no RCA. When both D-Gal and L-Trp were added, the incoming TrpR replaced the released GalR to bind circular template, resulting in an unchanged RCA inhibition. Therefore, an ANDN gate was constructed successfully (Fig. 4A). Finally, we parallel connected GalR and TrpR modules by mixing their individual circular templates together. In the presence of GalR and TrpR, using D-Gal and L-Trp as inputs, we found only when L-Trp was added in the absence of D-Gal, can RCA be inhibited completely. In other cases, RCA gave a ‘1’ output. Thus, an ORN gate was presented obviously (Fig. 4B).

### Constructing the other six two-input logic gates

**Figure 5:**
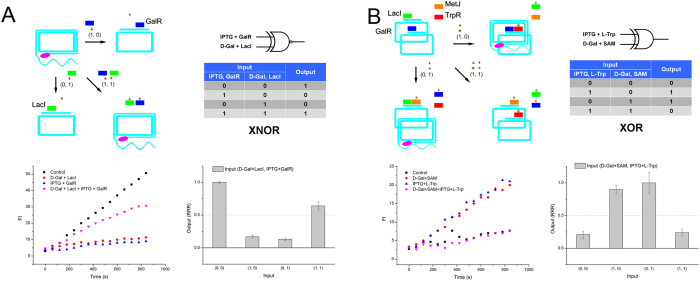
(**A**) XNOR gate of repressor-RCA, schematic, truth table, RCA curve and rate column. (**B**) XOR gate of repressor-RCA, schematic, truth table, RCA curve and rate column. Green rectangle: LacI; blue rectangle: GalR; orange rectangle: MetJ; red rectangle: TrpR; brown triangle: IPTG; brown diamond: D-Gal; brown exploding star: SAM; brown star: L-Trp; pink oval: DNA polymerase.

After accomplishing six two-input logic gates through “series” and “parallel” connections, we further constructed the other six two-input logic gates by using a more flexible combination. An XNOR gate is characterized by the condition in which two inputs have the same value, the outputs are ‘1’, and when inputs bear different values, the outputs are ‘0’. To realize XNOR gate, we designed a series connected inducer-regulated modules (LacI and GalR). The input A was the combination of GalR and IPTG, and the input B was the combination of LacI and D-Gal. When no inputs were added, RCA was carried out efficiently. When input A was added, GalR bound circular template to inhibit RCA. When input B was added, LacI bound circular template to suppress RCA. In the case that both inputs were added, the bound GalR and LacI were regulated by D-Gal and IPTG respectively, and both repressors released template to produce the RCA signal, therefore giving a ‘1’ output (Fig. 5A).

An XOR gate has inverse values of the XNOR gate. Here, we series connected LacI and MetJ modules, and also series connected GalR and TrpR modules. The two series modules were then parallel connected by mixing these two templates together. The four repressors were added as an initial state. We used the combination of IPTG and L-Trp as input A, and the combination of D-Gal and SAM as input B. Consequently, when no input was added, LacI and GalR bound both of the two templates, inhibiting RCA completely. When input A was added, IPTG induced LacI module to release RCA, though L-Trp triggered TrpR to bind another template along with GalR. When input B was added, GalR was regulated by D-Gal to free template, and gave a ‘1’ output. At the same time, SAM triggered MetJ to bind another template together with LacI. When both inputs were added, though LacI and GalR were free from templates by their inducers, MetJ and TrpR occupied the two templates with the help of their anti-inducers, therefore presented a ‘0’ output (Fig. 5B). Therefore, the XOR gate was successfully constructed.

**Figure 6:**
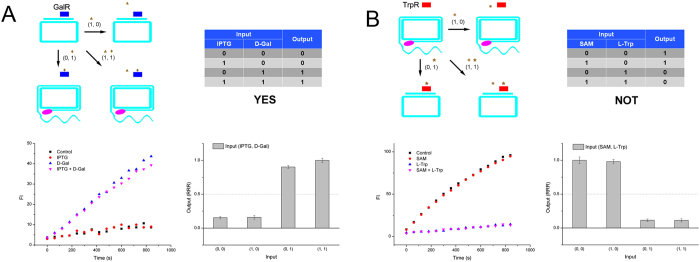
(**A**) YES gate of repressor-RCA, schematic, truth table, RCA curve and rate column. (**B**) NOT gate of repressor-RCA, schematic, truth table, RCA curve and rate column. Blue rectangle: GalR; red rectangle: TrpR; brown triangle: IPTG; brown diamond: D-Gal; brown exploding star: SAM; brown star: L-Trp; pink oval: DNA polymerase.

**Figure 7:**
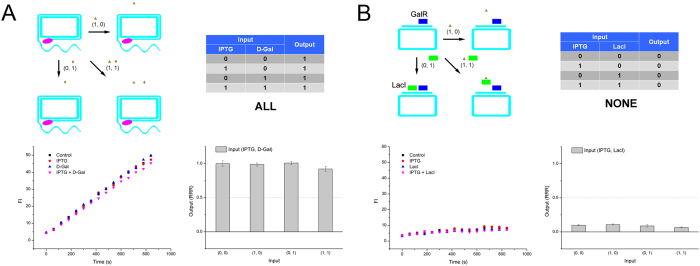
(**A**) ALL gate of repressor-RCA, schematic, truth table, RCA curve and rate column. (**B**) NONE gate of repressor-RCA, schematic, truth table, RCA curve and rate column. Green rectangle: LacI; blue rectangle: GalR; brown triangle: IPTG; brown diamond: D-Gal; pink oval: DNA polymerase.

In addition, by using a series LacI and GalR modules, with only one repressor (GalR) added, the two inducer inputs behaved a YES gate. As shown in Fig. 6A, only in case that input D-Gal was added, the GalR released circular template, and resulted in a ‘1’ output. Moreover, we series connected MetJ and TrpR modules in the presence of only one repressor (TrpR). Using two anti-inducers as inputs, only when no L-Trp was added, the logic gate gave a ‘1’ output, which was a typical NOT gate (Fig. 6B). Furthermore, by series connecting LacI and GalR modules, in the absence of both repressors, the two inducer inputs presented an ALL gate. Since, the RCA along the circular template was not affected by the inputs at all (Fig. 7A). Finally, when the series LacI and GalR modules in the presence of GalR repressor, and IPTG and LacI were added as inputs, the circular template was occupied by GalR despite the inputs values. Therefore, we acquired a NONE gate (Fig. 7B).

### Three input-logic gate constituted by basic modules

**Figure 8:**
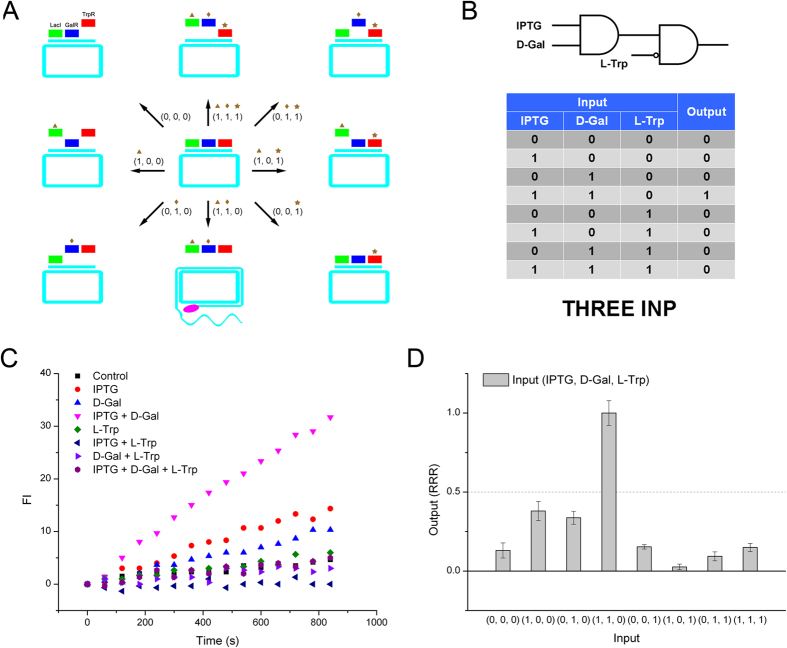
Three-input gate constituted by basic logic modules. (**A**) Schematic of the three-input gate of repressor-RCA. (**B**) Truth table of the three-input gate. (**C**) RCA fluorescence curve of the gate. (**D**) Relative RCA rate of the gate. Green rectangle: LacI; blue rectangle: GalR; orange rectangle: MetJ; red rectangle: TrpR; brown triangle: IPTG; brown diamond: D-Gal; brown exploding star: SAM; brown star: L-Trp; pink oval: DNA polymerase.

Multi-responsive logic gates need the flexibility to expand the connection of different logic modules. Here, we used a three-input logic gate model to illustrate that more basic modules can be connected in a complex way to facilitate a multiple inputs computations. In this section, we series connected three modules (LacI, GalR and TrpR) by designing their recognition sequences in series in one circular template. After adding all these three repressors, an initial state (0, 0, 0) was prepared. IPTG, D-Gal and L-Trp were selected as three inputs. From schematic, we can figure out that only IPTG and D-Gal were present without L-Trp, the circular template was free to employ RCA, and gave a ‘1’ output. In other cases, the output was ‘0’ (Fig. 8A). The RCA fluorescence curve and the output column plot were well in accord with the truth table (Fig. 8B–D). This three-input example demonstrated the potential of series and parallel connecting more modules to accomplish multiple inputs and more complex logic computations by this RCA-organized and RCA-output system. Given numerous allosteric TFs with their various regulating metabolites in nature, circular templates provide a flexible assembling platform in an easy way for logic module computation.

All the twelve two-input logic gates and three-input logic gate were constructed from four basic logic modules. Therefore, theoretically the thirteen logic gates can be derived from basic logic modules by formal proof of propositional calculus. To simplify the process, we used only three logical connectives: negation (¬), conjunction (∧) and disjunction (∨). The twelve two-input logic gates were expressed by propositional formula by using two atomic propositions (*P*, *Q*) connected by three connectives. Then, by employing “conjunction” (series) and “disjunction” (parallel) calculations of the four basic logic modules (ORN1, ORN2, NAND1, NAND2) and by substitutions, we proved all the thirteen constructed logic gates, and verified the truth tables in accord with the experiment results (See Supporting Information).

TF-regulated logic modules have been mainly employed in gene transcriptional circuits inside cells^7^,^9^,^12^. Though a cell system is superior in self-replication and robustness, the major concern is its unpredictable complex intracellular biomolecule networks. Therefore, cell-free *in vitro* expression systems attracted increasing interests for logic circuits construction^40^,^41^,^42^. Here, the repressor-RCA system provided another novel type of TF-regulated logic circuits. The most distinct advantage of this system is its convenience, such as time-saving and few steps. It also has the ability to construct series and parallel logic connections. However, for layered circuits, it still needs to be improved in the future. Detailed comparison of these different TF-regulated logic circuits was listed in Table S1 (See supporting information).

In summary, we have established two pairs of basic logic modules based on repressor-RCA principle for the first time. Moreover, by using circular templates as assembling platform, we constructed twelve two-input logic gates and one three-input logic gate by “series” and “parallel” connection of the four basic logic modules. This module assembling derivation was further verified by propositional calculus. Despite the flexibly assembled repressors by RCA circular templates, this logic system provided a fast and convenient output form of RCA signal rather than gene expression in cells. With the advent of more and more discovered orthogonal TFs^8^, this system can employ more TF candidates in the fields of molecular computing. It is also helpful for developing TF-based metabolites sensing methods.

## Methods

### Chemicals, oligonucleotides and tool enzymes

IPTG, D-Galactose, SAM and L-Trp were from Sigma-Aldrich (Darmstadt, Germany). SYBR Green II was from Invitrogen (Waltham, MA). Tryptone and Yeast extract were from Oxoid LTD. (Basingstoke, England). Oligonucleotides of HPLC grade were from Genscript Corporation (Nanjing, China). The sequences were listed in Tables S2 and S3. Their concentrations were determined by UV absorption at 260 nm. Oligonucleotides used for circularization were phosphorylated at the 5′-terminal. Repressor recognition sequences embedded in circular templates were listed in Table S4.

Phi29 DNA polymerase was from NEB (Beijing, China). Pfu DNA polymerase was from Tiangen Biotech (Beijing, China). Restriction endonucleases (EcoRI, HindIII and XhoI), T4 DNA ligase and DNA ligation kit were from TaKaRa Biotechnology Co. Ltd. (Dalian, China). DNA Gel Extraction Kit and Plasmid Miniprep Kit were from Axygen Scientific Inc. (Union City, CA). Ni-NTA column was from GE Healthcare (Buckinghamshire, UK). pET28a vector, DH5α and BL21 strain were kept in our lab.

### Cloning, expression and purification of the three repressors

LacI, GalR and MetJ genes were obtained through PCR amplification from *E. coli* strain DH5α. The primer pairs were designed containing restriction endonuclease (RE) recognition sequences (EcoRI and HindIII for LacI and MetJ cloning, EcoRI and XhoI for GalR cloning). Primer sequences were listed in Table S1. PCR products of LacI were 1099 bp, products of GalR were 1050 bp, and MetJ were 336 bp. The PCR products were cleaved by their corresponding REs at 37 °C for 2 h. The cleaved products were purified by agarose gel electrophoresis and gel extraction purification. The three digested gene fragments and corresponding digested pET28a plasmid were ligated by T4 DNA ligase at 16 °C overnight. Recombinant plasmids were transformed into competent DH5α cells, which were cultured in Luria-Bertani (LB) plate containing kanamycin (30 μg/mL). Clones were picked and cultured in kanamycin LB media, and the extracted plasmids were identified by REs double digestion (EcoRI/HindIII for LacI and MetJ cloning, EcoRI/XhoI for GalR cloning). The positive recombinant plasmids were further confirmed by DNA sequencing.

Recombinant plasmids of the three genes were transformed into competent BL21 cells for protein expression. Transformed cells were cultured in LB media containing kanamycin. When OD_600_ of cell culture reached 0.6, BL21 cells were induced by 5 mM isopropyl β-*D*-1-Thiogalactopyranoside (IPTG) at 37 °C for 4 hours. The cells were collected by centrifugation at 4 °C.

For protein purification, the centrifuged BL21 cells were re-suspended in lysis buffer. After cells were ultrasonicated, cell lysates were centrifuged at 4 °C. The supernatant was purified by Ni-NTA column by following the operation manual. Briefly, after supernatant flowthrough, the column was washed by at least 10 volumns of washing buffer and then was eluted by 500 mM imidazole. The protein solution was prepared in 20 mM PBS pH 8.0 after dialyzation at 4 °C and was stored at −20 °C. The purified protein was verified by sodium dodecyl sulfate-polyacrylamide gel electrophoresis (SDS-PAGE). Protein concentration was determined by Bradford method.

### Circularization and RCA reaction

For circularization, The 5′- and 3′-portions of the circular template (1 μM) were hybridized with a splint oligonucleotide (1 μM) in a head-to-tail way, and then were ligated by T4 DNA ligase (175 U) in ligation buffer containing 50 mM Tris pH 8.0, 10 mM MgCl_2_, 5 mM DTT and 0.1 mM ATP at 16 °C for 60 min.

RCA reaction solution was composed of 0.05 pmol ligated circular template, 50 mM Tris, 10 mM MgCl_2_, 10 mM (NH_4_)_2_SO_4_, 4 mM DTT (pH 7.5), SYBR Green II (1:10000) and phi29 DNA polymerase 3 U in 100 μL. The repressor (5 pmol LacI, 10 pmol GalR, 5 pmol MetJ, and 10 pmol TrpR) were added or not added according to different logic gates operation (Tables S5~S6). Fluorescence signals were recorded with excitation wavelength at 480 nm and emission wavelength at 524 nm by Microplate Reader (Infinite M200, Tecan, USA). The fluorescence signals were real-time collected for over ~15 min at 37 °C, and were used to plot RCA fluorescence curve. The RCA rate was calculated as the slope of the linear part of the RCA fluorescence curve, and was normalized to be relative RCA rate (RRR) for easy comparison.

## Additional Information

**How to cite this article**: Wei, H. *et al*. Repressor logic modules assembled by rolling circle amplification platform to construct a set of logic gates. *Sci. Rep.* **6**, 37477; doi: 10.1038/srep37477 (2016).

**Publisher’s note:** Springer Nature remains neutral with regard to jurisdictional claims in published maps and institutional affiliations.

## Supplementary Material

Supplementary Information

## Figures and Tables

**Figure 1:**
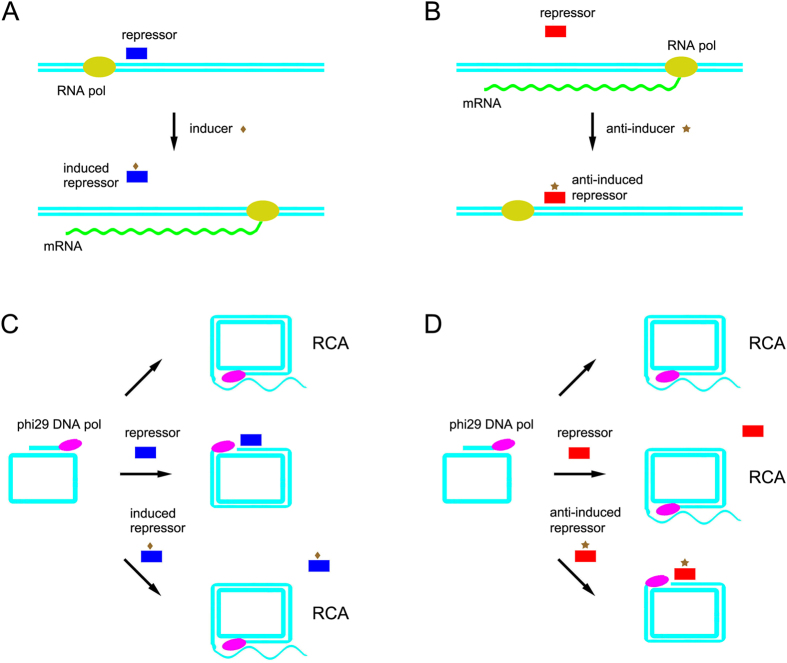
Schematic of two types of repressor-RCA. (**A**) Inducer-regulated RNA transcription *in vivo*. (**B**) Anti-inducer-regulated RNA transcription *in vivo*. (**C**) Inducer-regulated repressor-RCA *in vitro*. (**D**) Anti-inducer-regulated repressor-RCA *in vitro*. Blue rectangle: repressor (GalR); red rectangle: repressor (TrpR); brown diamond: inducer (D-Gal); brown star: anti-inducer (L-Trp); cyan straight line: DNA; green wave line: mRNA; golden oval: RNA polymerase; pink oval: DNA polymerase.
