# Supplementary material for: Repressor logic modules assembled by rolling circle amplification platform to construct a set of logic gates
Source: Sci Rep. 2016 Nov 21;6:37477. doi: 10.1038/srep37477 (PMC5116584; doi:10.1038/srep37477)
Supplement: Supplementary Information [file srep37477-s1.doc]

Supplementary information

**Repressor logic modules assembled by rolling circle amplification platform to construct a set of logic gates**

Hua Wei^1,2^, Bo Hu^2^, Suming Tang^2^, Guojie Zhao^2,*^ & Yifu Guan^2,*^

^1^Animal Science and Veterinary Medicine College, Shenyang Agricultural University, #120 Dongling Road, Shenyang, Liaoning, 110866, China

^2^Department of Biochemistry and Molecular Biology, China Medical University, #77 Puhe Road, Shenyang, Liaoning, 110122, China

^*^Correspondance and requests for materials should be addressed to G.Z. ([gjzhao@mail.cmu.edu.cn](mailto:gjzhao@mail.cmu.edu.cn)); Y.G. ([yfguan@mail.cmu.edu.cn](mailto:yfguan@mail.cmu.edu.cn))

*Supplementary information*

**Supplementary Table S1.** Comparison of the three TF-regulated logic gates systems.

|  | *In vivo* gene transcriptional modules | *In vitro* gene transcriptional modules | *In vitro* RCA modules |
| --- | --- | --- | --- |
| Signal processing from input to output | RNA transcription and protein translation | RNA transcription and protein translation | DNA replication |
| TF switch control location | Transcription | Transcription | Replication |
| Requisite basic materials | Recombinant plasmids and cells | Recombinant plasmids and cell extracts | Circular DNA templates and purified TFs |
| General procedure | 1. Plasmid transformation 2. Plate cell culture 3. Single clone culture 4. Inducing culture 5. Fluorescence detection | 1. Plasmid transcription and translation reactions 2. Fluorescence detection | 1. Repressor-RCA 2. Fluorescence detection |
| Reaction time | 2 ~ 3 days | Several hours | ~ 30 min |
| Ease of use | Laborious | Easy | Very easy |
| Main cost | Plasmid construction and cell culture | Plasmid construction and cell-free expression system | TF purification |
| Flexibility for series and parallel connection | Yes | Yes | Yes |
| Flexibility for layered circuits | Strong | Strong | Weak |
| Precise control of reaction content | No | No | Yes |
| Robustness against external environment | Strong | Weak | Weak |
| References | [^1-4^](#_ENREF_1) | [^5-7^](#_ENREF_5) | This article |


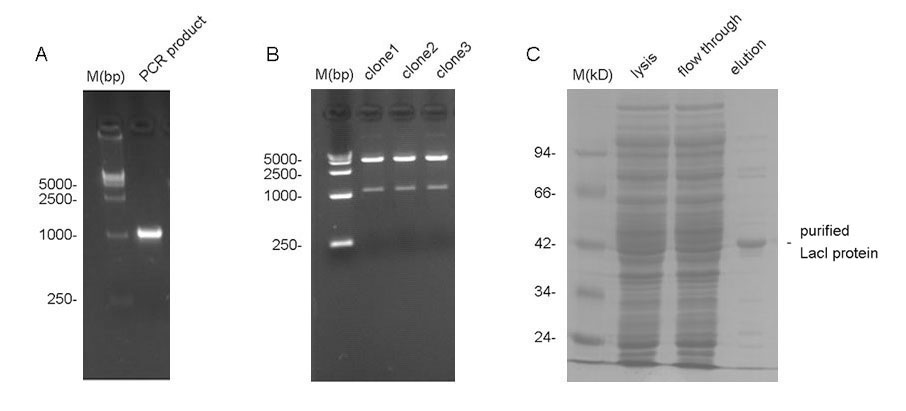


**Supplementary Figure S1.** Cloning, expression and purification of LacI protein. (A) Agarose gel electrophoresis of PCR product of LacI gene. (B) Agarose gel electrophoresis analysis of RE double digestion of recombinant pET28a-LacI plasmid. (C) SDS-PAGE analysis of the purification process of LacI protein.


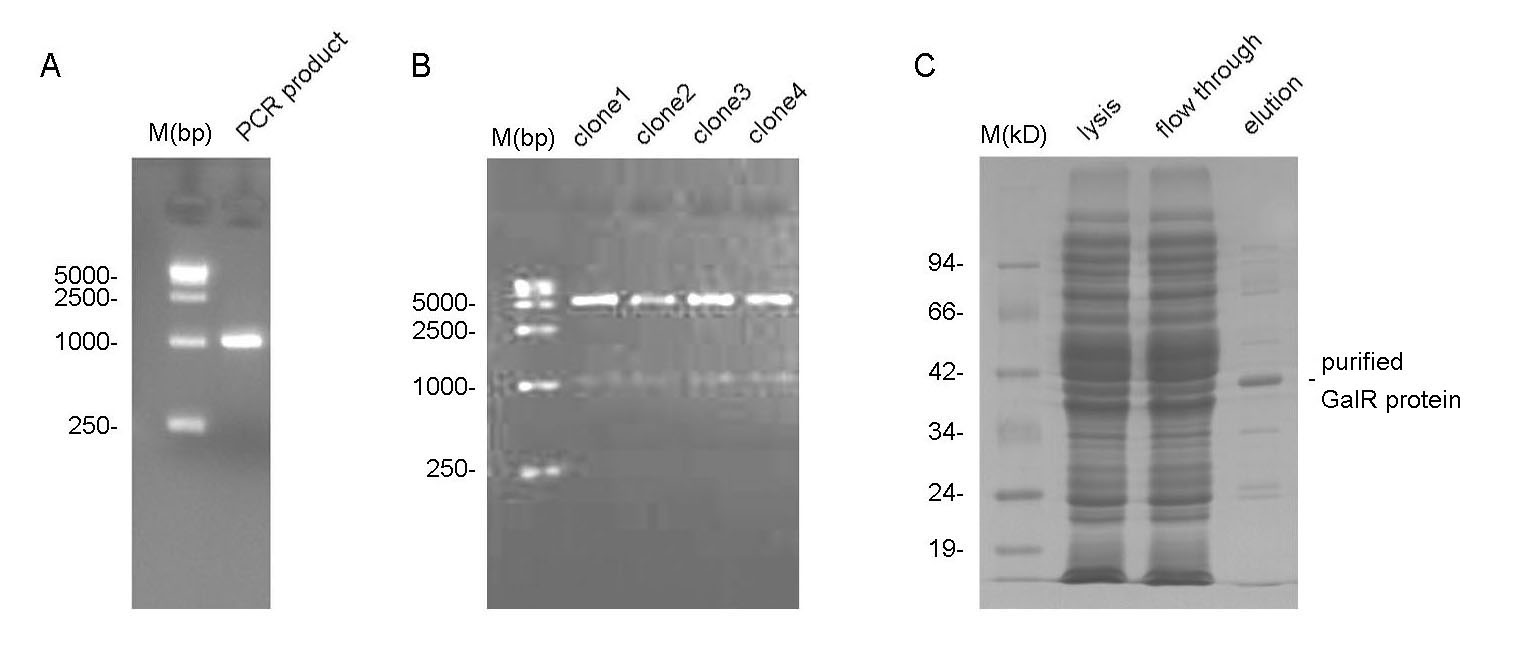


**Supplementary Figure S2.** Cloning, expression and purification of GalR protein. (A) Agarose gel electrophoresis of PCR product of GalR gene. (B) Agarose gel electrophoresis analysis of RE double digestion of recombinant pET28a-GalR plasmid. (C) SDS-PAGE analysis of the purification process of GalR protein.


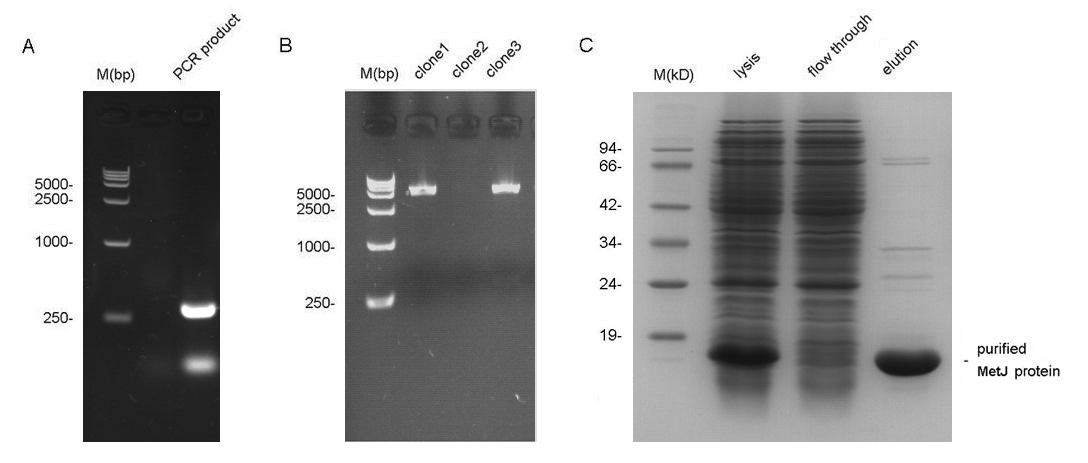


**Supplementary Figure S3.** Cloning, expression and purification of MetJ protein. (A) Agarose gel electrophoresis of PCR product of MetJ gene. (B) Agarose gel electrophoresis analysis of RE double digestion of recombinant pET28a-MetJ plasmid. (C) SDS-PAGE analysis of the purification process of MetJ protein.


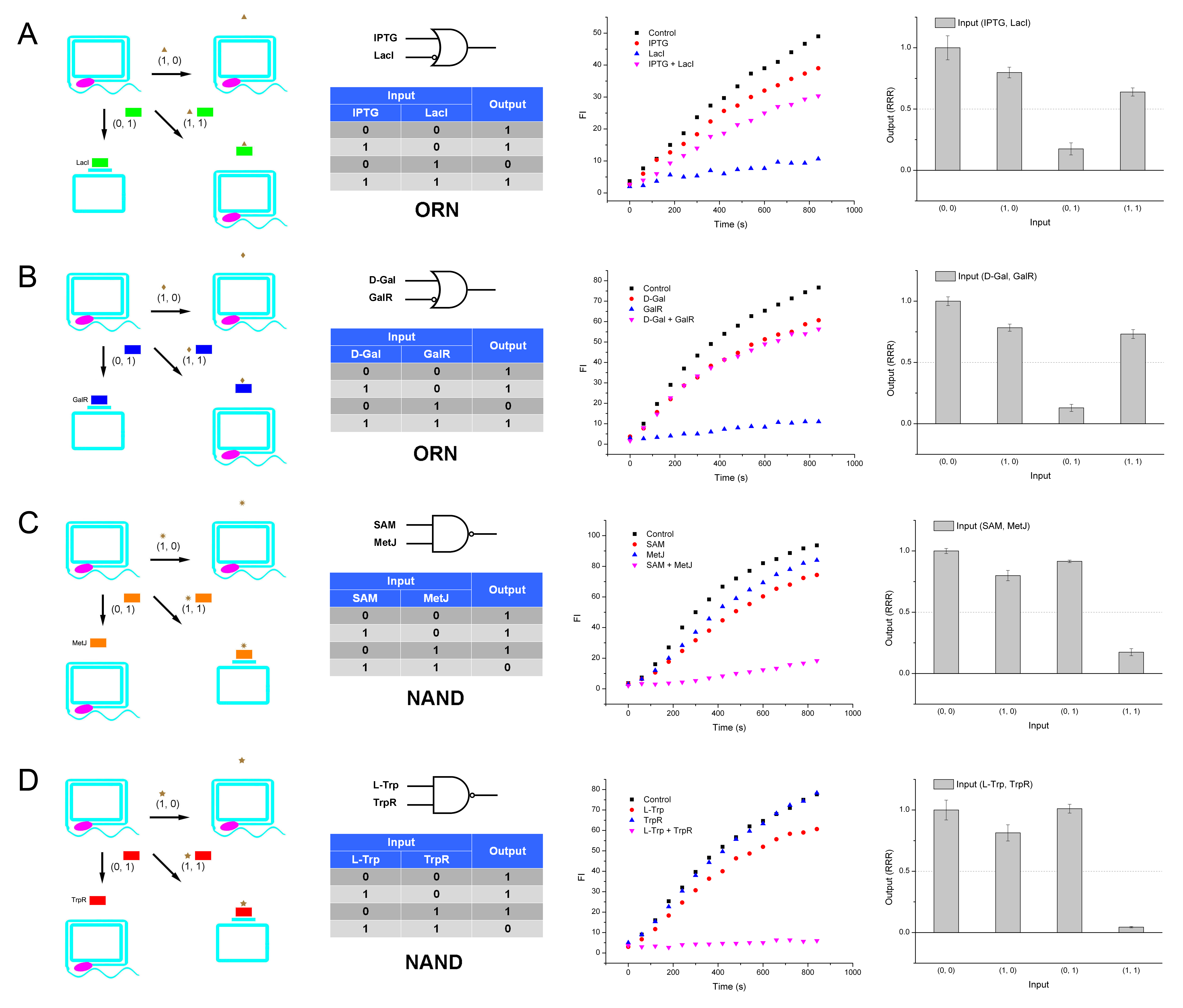


**Supplementary Figure S4.** Two pairs of basic repressor-RCA logic modules. (A) ORN gate of IPTG-LacI regulated RCA, schematic, truth table, RCA curve and rate column. (B) ORN gate of D-Gal-GalR regulated RCA, schematic, truth table, RCA curve and rate column. (C) NAND gate of SAM-MetJ regulated RCA, schematic, truth table, RCA curve and rate column. (D) NAND gate of L-Trp-TrpR regulated RCA, schematic, truth table, RCA curve and rate column. Green rectangle: LacI; blue rectangle: GalR; orange rectangle: MetJ; red rectangle: TrpR; brown triangle: IPTG; brown diamond: D-Gal; brown exploding star: SAM; brown star: L-Trp; pink oval: DNA polymerase.


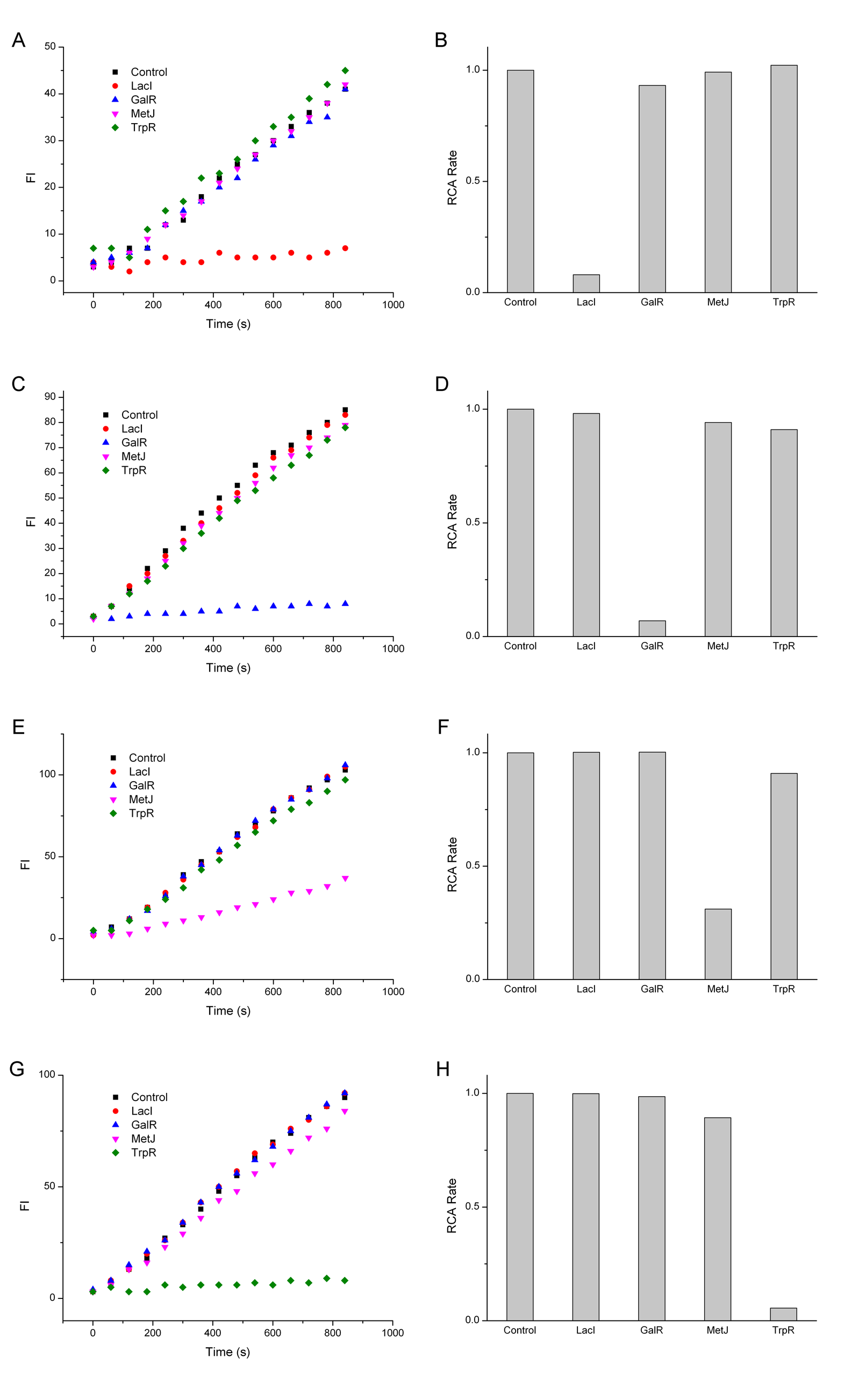


**Supplementary Figure S5.** Sequence binding specificity of the four repressors. (A) RCA fluorescence curve of LacI recognition sequence affected by four repressors. (B) RCA rates of LacI recognition sequence affected by four repressors. (C) RCA fluorescence curve of GalR recognition sequence affected by four repressors. (D) RCA rates of GalR recognition sequence affected by four repressors. (E) RCA fluorescence curve of MetJ recognition sequence affected by four repressors. (F) RCA rates of MetJ recognition sequence affected by four repressors. (G) RCA fluorescence curve of TrpR recognition sequence affected by four repressors. (H) RCA rates of TrpR recognition sequence affected by four repressors.


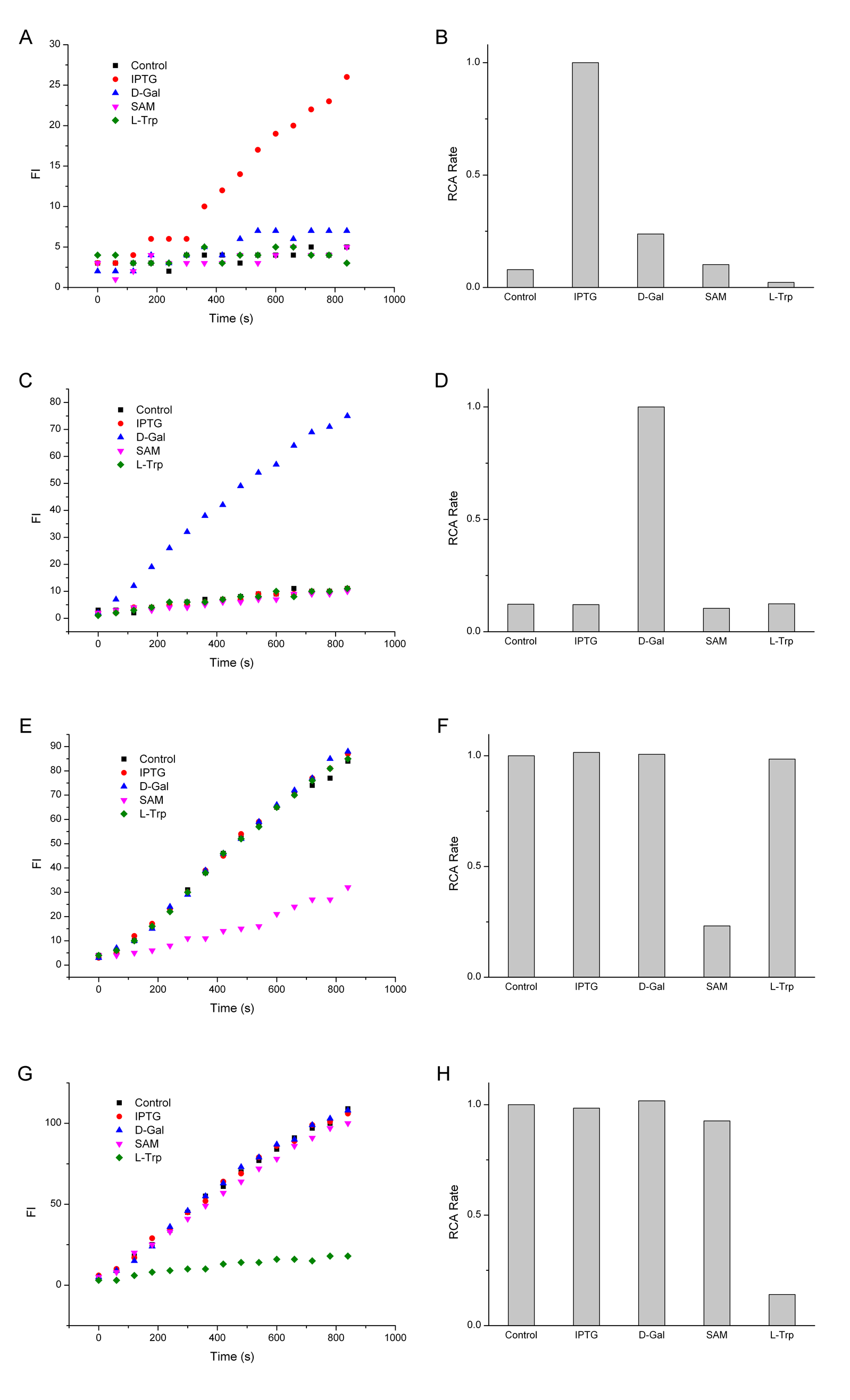


**Supplementary Figure S6.** Allosteric regulation specificity of repressors by four metabolites. (A) RCA fluorescence curve of circular template regulated by LacI affected by four metabolites. (B) RCA rates of circular template regulated by LacI affected by four metabolites. (C) RCA fluorescence curve of circular template regulated by GalR affected by four metabolites. (D) RCA rates of circular template regulated by GalR affected by four metabolites. (E) RCA fluorescence curve of circular template regulated by MetJ affected by four metabolites. (F) RCA rates of circular template regulated by MetJ affected by four metabolites. (G) RCA fluorescence curve of circular template regulated by TrpR affected by four metabolites. (H) RCA rates of circular template regulated by TrpR affected by four metabolites.


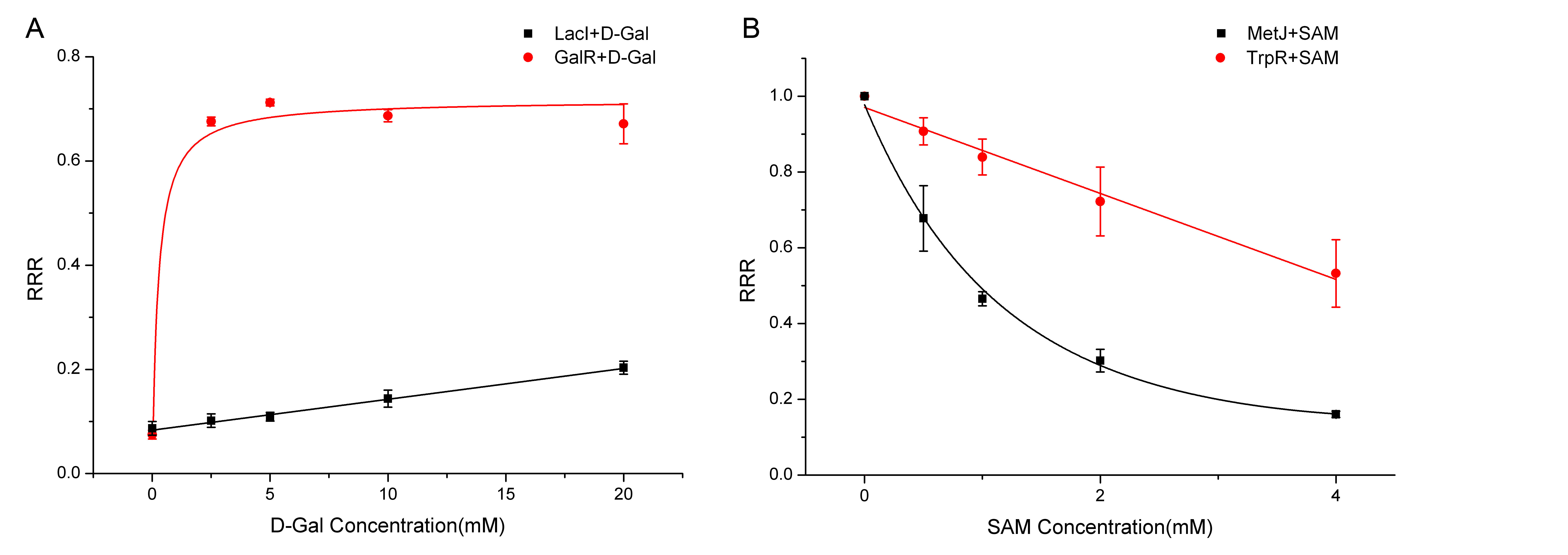


**Supplementary Figure S7.** Comparison of repressor-RCA rates affected by cross-reacted metabolites of different concentrations. (A) Comparison of LacI and GalR induced by different concentrations of D-Gal respectively. (B) Comparison of MetJ and TrpR anti-induced by different concentrations of SAM respectively.

**Supplementary Table S2.** Primers used for cloning repressors.

| Repressor | Primer | Sequence* |
| --- | --- | --- |
| LacI | Upstream | 5'-AAGAATTCGTGAAACCAGTAACGCTATACGATG-3' |
|  | Downstream | 5'-ACAAGCTTTCACTGCCCGCTTTCCAGTC-3' |
| GalR | Upstream | 5'-TCCGAATTCATGGCGACCATAAAGGATGT-3' |
|  | Downstream | 5'-GTGCTCGAGTTAGTCGCTGGTTGCATGAT-3' |
| MetJ | Upstream | 5'-TTAGAATTCATGGCTGAATGGAGCGGC-3' |
|  | Downstream | 5'-TCCAAGCTTTTAGTATTCCCACGTCTCCG-3' |

*The underlined sequences are recognition sequences of restriction endonucleases EcoRI (GAATTC), XhoI (CTCGAG) and HindIII (AAGCTT).

**Supplementary Table S3.** Oligonucleotides for RCA used in this study.

| Name | Sequence* |
| --- | --- |
| CT-lac | 5'-AATT-TATGATTAGTTTCCTTCCTGTTTCATCCAGGAACTTGC-  -AATTGTTATCCGCTCAC-3' |
| SP-lac | 5'-TAATCATA-AATTGTGAGCGGATAACAATT-GCAAGTTC-3' |
| CT-mgl | 5'-GGTTACAT-GATGATTACTTTCCTGCCTGTGTATTTATCCAAGAACTTCC-  -TTGAAAGC-3' |
| SP-mgl | 5'-TAATCATC-ATGTAACCGCTTTCAA-GGAAGTTC-3' |
| CT-met | 5'-TCTAGACGTCT-ATGCTTGCTTTAATGCCTGTGCTTTTATCCAGGAACTTC-  -AGACG-3' |
| SP-met | 5'-CAT-AGACGTCTAGACGTCT-GAAGT-3' |
| CT-trp | 5'-TAGTTC-GATGATTAGTTTCCTGCCTGTGCTTTTATCCAGGAACTTGC-  -GTACTAGTTAAC-3' |
| SP-trp | 5'-TAATCATC-GAACTAGTTAACTAGTAC-GCAAGTTC-3' |
| CT-mgl-lac | 5'-GGTTACAT-GATGATTA-AATTGTGAGCGGATAACAATT-  -CTTTCCTGCCTGTGTATTTATCCAAGAACTTCC-TTGAAAGC-3' |
| SP-mgl-lac | 5'-AAAG-AATTGTTATCCGCTCACAATT-TAATCATC-  -ATGTAACCGCTTTCAA-GGAAGTTC-3' |
| CT-met-trp | 5'-GTCTAGACGTCT-AATCATTT-GTACTAGTTAACTAGTTC-  -CTTTCCTGCCTGTGTATTTATCCAAGAACTTTC-AGAC-3' |
| SP-met-trp | 5'-GAAAG-GAACTAGTTAACTAGTAC-AAATGATT-  -AGACGTCTAGACGTCT-GAAAGTTC-3' |
| CT-mgl-trp | 5'-GGTTACAT-GATGATTA-GTACTAGTTAACTAGTTC-  -CTTTCCTGCCTGTGTATTTATCCAAGAACTTCC-TTGAAAGC-3' |
| SP-mgl-trp | 5'-GAAAG-GAACTAGTTAACTAGTAC-TAATCATC-  -ATGTAACCGCTTTCAA-GGAAGTTC-3' |
| CT-lac-met | 5'-AATT-TATGATTA-AGACGTCTAGACGTCT-  -GTTTCCTTCCTGTTTCATCCAGGAACTTGC-AATTGTTATCCGCTCAC-3' |
| SP-lac-met | 5'-AAAC-AGACGTCTAGACGTCT-TAATCATA-  -AATTGTGAGCGGATAACAATT-GCAAGTTC-3' |
| CT-mlt | 5'-TGAGCGGATAACAATT-GATTATTA-GTACTAGTTAACTAGTTC-  -GTTTCCTTCCTGTTTCATCCAGGAAATTGC-TTGAAAGCGGTTACAT-  -GATATTA-AATTG-3' |
| SP-mlt | 5'-AAAC-GAACTAGTTAACTAGTAC-TAATAATC-AATTGTTATCCGCTCACAATT-  -TAATATC-ATGTAACCGCTTTCAA-GCAATTT-3' |

*The underlined sequences are repressor-binding sequences.

**Supplementary Table S4.** Recognition sequences of repressors used in this study.

| Repressor | Recognition sequence | Reference |
| --- | --- | --- |
| LacI | 5'-AATTGTGAGCGGATAACAATT-3' | [^8^](#_ENREF_8) |
| GalR | 5'-ATGTAACCGCTTTCAA-3' | [^9^](#_ENREF_9) |
| MetJ | 5'-AGACGTCTAGACGTCT-3' | [^10^](#_ENREF_10) |
| TrpR | 5'-GAACTAGTTAACTAGTAC-3' | [^11^](#_ENREF_11) |

**Supplementary Table S5.** Four basic logic modules.

| Logic Gates | Circular templates | Input A | Input B |
| --- | --- | --- | --- |
| ORN | CT-lac | IPTG | LacI |
| ORN | CT-mgl | D-Gal | GalR |
| NAND | CT-met | SAM | MetJ |
| NAND | CT-trp | L-Trp | TrpR |

**Supplementary Table S6.** Twelve two-input gates constituted by repressor modules.

| Logic Gates | Circular templates | Repressors | Input A | Input B |
| --- | --- | --- | --- | --- |
| AND | CT-mgl-lac | LacI, GalR | IPTG | D-Gal |
| OR | CT-lac, CT-mgl | LacI, GalR | IPTG | D-Gal |
| NOR | CT-met-trp | MetJ, TrpR | SAM | L-Trp |
| NAND | CT-met, CT-trp | MetJ, TrpR | SAM | L-Trp |
| ANDN | CT-mgl-trp | GalR, TrpR | D-Gal | L-Trp |
| ORN | CT-mgl, CT-trp | GalR, TrpR | D-Gal | L-Trp |
| XNOR | CT-mgl-lac | −− | IPTG+GalR | D-Gal+LacI |
| XOR | CT-mgl-trp, CT-lac-met | LacI, GalR, MetJ, TrpR | IPTG+L-Trp | D-Gal+SAM |
| YES | CT-mgl-lac | GalR | IPTG | D-Gal |
| NOT | CT-met-trp | TrpR | SAM | L-Trp |
| ALL | CT-mgl-lac | −− | IPTG | D-Gal |
| NONE | CT-mgl-lac | GalR | IPTG | LacI |

**Propositional calculus derivation of thirteen logic gates from two pairs of basic logic modules:**

Propositions: *P*, *Q*

Logical connectives:

Negation: ¬

Conjunction: ∧

Disjunction: ∨

Propositional formula of twelve two-input logic gates:

AND gate: *P* ∧ *Q*

OR gate: *P* ∨ *Q*

NOR gate: ¬ *P* ∧ ¬ *Q*

NAND gate: ¬ *P* ∨ ¬ *Q*

ANDN gate: *P* ∧ ¬ *Q*

ORN gate: *P* ∨ ¬ *Q*

XNOR gate: (¬ *P* ∨ *Q*) ∧ (*P* ∨ ¬ *Q*)

XOR gate: (¬ *P* ∧ *Q*) ∨ (*P* ∧ ¬ *Q*)

YES gate: *P*

NOT gate: ¬ *P*

ALL gate: 1

NONE gate: 0

Define propositions:

M1 = IPTG; R1 = LacI;

M2 = D-Gal; R2 = GalR;

M’1 = SAM; R’1 = MetJ;

M’2 = L-Trp; R’2 = TrpR;

**Two pairs of basic logic modules:**

ORN1:

*P* ∨ ¬ *Q* ⇔ M1 ∨ ¬ R1

ORN2:

*P* ∨ ¬ *Q* ⇔ M2 ∨ ¬ R2

NAND1:

¬ *P* ∨ ¬ *Q* ⇔ ¬ M’1 ∨ ¬ R’1

NAND2:

¬ *P* ∨ ¬ *Q* ⇔ ¬ M’2 ∨ ¬ R’2

**Two-input and three-input logic gates deduced from basic logic modules:**

AND gate (series connecting two ORN)

Given:

R1, R2 = 1

Then:

ORN1 ∧ ORN2

⇔ (M1 ∨ ¬ R1) ∧ (M2 ∨ ¬ R2)

⇔ (M1 ∨ ¬ 1) ∧ (M2 ∨ ¬ 1)

⇔ M1 ∧ M2

Truth table:

| **Input** | | **Output** |
| --- | --- | --- |
| **M1** | **M2** |
| **0** | **0** | **0** |
| **1** | **0** | **0** |
| **0** | **1** | **0** |
| **1** | **1** | **1** |

OR gate (parallel connecting two ORN)

Given:

R1, R2 = 1

Then:

ORN1 ∨ ORN2

⇔ (M1 ∨ ¬ R1) ∨ (M2 ∨ ¬ R2)

⇔ (M1 ∨ ¬ 1) ∨ (M2 ∨ ¬ 1)

⇔ (M1) ∨ (M2)

⇔ M1 ∨ M2

Truth table:

| **Input** | | **Output** |
| --- | --- | --- |
| **M1** | **M2** |
| **0** | **0** | **0** |
| **1** | **0** | **1** |
| **0** | **1** | **1** |
| **1** | **1** | **1** |

NOR gate (series connecting two NAND)

Given:

R’1, R’2 = 1

Then:

NAND1 ∧ NAND2

⇔ (¬ M’1 ∨ ¬ R’1) ∧ (¬ M’2 ∨ ¬ R’2)

⇔ (¬ M’1 ∨ ¬ 1) ∧ (¬ M’2 ∨ ¬ 1)

⇔ (¬ M’1) ∧ (¬ M’2)

⇔ ¬ M’1 ∧ ¬ M’2

Truth table:

| **Input** | | **Output** |
| --- | --- | --- |
| **M’1** | **M’2** |
| **0** | **0** | **1** |
| **1** | **0** | **0** |
| **0** | **1** | **0** |
| **1** | **1** | **0** |

NAND gate (parallel connecting two NAND)

Given:

R’1, R’2 = 1

Then:

NAND1 ∨ NAND2

⇔ (¬ M’1 ∨ ¬ R’1) ∨ (¬ M’2 ∨ ¬ R’2)

⇔ (¬ M’1 ∨ ¬ 1) ∨ (¬ M’2 ∨ ¬ 1)

⇔ (¬ M’1) ∨ (¬ M’2)

⇔ ¬ M’1 ∨ ¬ M’2

Truth table:

| **Input** | | **Output** |
| --- | --- | --- |
| **M’1** | **M’2** |
| **0** | **0** | **1** |
| **1** | **0** | **1** |
| **0** | **1** | **1** |
| **1** | **1** | **0** |

ANDN gate (series connecting ORN and NAND)

Given:

R2, R’2 = 1

Then:

ORN2 ∧ NAND2

⇔ (M2 ∨ ¬ R2) ∧ (¬ M’2 ∨ ¬ R’2)

⇔ (M2 ∨ ¬ 1) ∧ (¬ M’2 ∨ ¬ 1)

⇔ (M2) ∧ (¬ M’2)

⇔ M2 ∧ ¬ M’2

Truth table:

| **Input** | | **Output** |
| --- | --- | --- |
| **M2** | **M’2** |
| **0** | **0** | **0** |
| **1** | **0** | **1** |
| **0** | **1** | **0** |
| **1** | **1** | **0** |

ORN gate (parallel connecting ORN and NAND)

Given:

R2, R’2 = 1

Then:

ORN2 ∨ NAND2

⇔ (M2 ∨ ¬ R2) ∨ (¬ M’2 ∨ ¬ R’2)

⇔ (M2 ∨ ¬ 1) ∨ (¬ M’2 ∨ ¬ 1)

⇔ (M2) ∨ (¬ M’2)

⇔ M2 ∨ ¬ M’2

Truth table:

| **Input** | | **Output** |
| --- | --- | --- |
| **M2** | **M’2** |
| **0** | **0** | **1** |
| **1** | **0** | **1** |
| **0** | **1** | **0** |
| **1** | **1** | **1** |

XNOR gate (series connecting two ORN)

Given:

M1 = R2; M2 = R1

Then:

ORN1 ∧ ORN2

⇔ (M1 ∨ ¬ R1) ∧ (M2 ∨ ¬ R2)

⇔ (M1,R2 ∨ ¬ M2,R1) ∧ (M2,R1 ∨ ¬ M1,R2)

Truth table:

| **Input** | | **Output** |
| --- | --- | --- |
| **M1, R2** | **M2, R1** |
| **0** | **0** | **1** |
| **1** | **0** | **0** |
| **0** | **1** | **0** |
| **1** | **1** | **1** |

XOR gate (series and parallel connecting ORN and NAND)

Given:

R1, R2, R1’, R2’ = 1; M1 = M’2; M2 = M’1

Then:

(ORN1 ∧ NAND2) ∨ (ORN2 ∧ NAND1)

⇔ ((M1 ∨ ¬ R1) ∧ (¬ M’1 ∨ ¬ R’1)) ∨ ((M2 ∨ ¬ R2) ∧ (¬ M’2 ∨ ¬ R’2))

⇔ ((M1 ∨ ¬ 1) ∧ (¬ M’1 ∨ ¬ 1)) ∨ ((M2 ∨ ¬ 1) ∧ (¬ M’2 ∨ ¬ 1))

⇔ ((M1) ∧ (¬ M’1)) ∨ ((M2) ∧ (¬ M’2))

⇔ (M1 ∧ ¬ M’1) ∨ (M2 ∧ ¬ M’2)

⇔ (M1,M’2 ∧ ¬ M2,M’1) ∨ (M2,M’1 ∧ ¬ M1,M’2)

Truth table:

| **Input** | | **Output** |
| --- | --- | --- |
| **M1, M’2** | **M2, M’2** |
| **0** | **0** | **0** |
| **1** | **0** | **1** |
| **0** | **1** | **1** |
| **1** | **1** | **0** |

YES gate (series connecting two ORN)

Given:

R1 = 0; R2 = 1

Then:

ORN1 ∧ ORN2

⇔ (M1 ∨ ¬ R1) ∧ (M2 ∨ ¬ R2)

⇔ (M1 ∨ ¬ 0) ∧ (M2 ∨ ¬ 1)

⇔ (M1 ∨ 1) ∧ (M2 ∨ 0)

⇔ (1) ∧ (M2)

⇔ M2

Truth table:

| **Input** | | **Output** |
| --- | --- | --- |
| **M1** | **M2** |
| **0** | **0** | **0** |
| **1** | **0** | **0** |
| **0** | **1** | **1** |
| **1** | **1** | **1** |

NOT gate (series connecting two NAND)

Given:

R’1 = 0; R’2 = 1

Then:

NAND1 ∧ NAND2

⇔ (¬ M’1 ∨ ¬ R’1) ∧ (¬ M’2 ∨ ¬ R’2)

⇔ (¬ M’1 ∨ ¬ 0) ∧ (¬ M’2 ∨ ¬ 1)

⇔ (¬ M’1 ∨ 1) ∧ (¬ M’2 ∨ 0)

⇔ (1) ∧ (¬ M’2)

⇔ ¬ M’2

Truth table:

| **Input** | | **Output** |
| --- | --- | --- |
| **M’1** | **M’2** |
| **0** | **0** | **1** |
| **1** | **0** | **1** |
| **0** | **1** | **0** |
| **1** | **1** | **0** |

ALL gate (series connecting two ORN)

Given:

R1, R2 = 0

Then:

ORN1 ∧ ORN2

⇔ (M1 ∨ ¬ R1) ∧ (M2 ∨ ¬ R2)

⇔ (M1 ∨ ¬ 0) ∧ (M2 ∨ ¬ 0)

⇔ (M1 ∨ 1) ∧ (M2 ∨ 1)

⇔ (1) ∧ (1)

⇔ 1

Truth table:

| **Input** | | **Output** |
| --- | --- | --- |
| **M1** | **M2** |
| **0** | **0** | **1** |
| **1** | **0** | **1** |
| **0** | **1** | **1** |
| **1** | **1** | **1** |

NONE GATE (series connecting two ORN)

Given:

M2 = 0; R2 = 1

Then:

ORN1 ∧ ORN2

⇔ (M1 ∨ ¬ R1) ∧ (M2 ∨ ¬ R2)

⇔ (M1 ∨ ¬ R1) ∧ (0 ∨ ¬ 1)

⇔ (M1 ∨ ¬ R1) ∧ 0

⇔ 0

Truth table:

| **Input** | | **Output** |
| --- | --- | --- |
| **M1** | **R1** |
| **0** | **0** | **0** |
| **1** | **0** | **0** |
| **0** | **1** | **0** |
| **1** | **1** | **0** |

Three-input gate (series connecting two ORN and one NAND)

Given:

R1, R2, R’2 = 1

Then:

(M1 ∨ ¬ R1) ∧ (M2 ∨ ¬ R2) ∧ (¬ M’2 ∨ ¬ R’2)

⇔ (M1 ∨ ¬ 1) ∧ (M2 ∨ ¬ 1) ∧ (¬ M’2 ∨ ¬ 1)

⇔ (M1) ∧ (M2) ∧ (¬ M’2)

⇔ M1 ∧ M2 ∧ ¬ M’2

Truth table:

| **Input** | | | **Output** |
| --- | --- | --- | --- |
| **M1** | **M2** | **M’2** |
| **0** | **0** | **0** | **0** |
| **1** | **0** | **0** | **0** |
| **0** | **1** | **0** | **0** |
| **1** | **1** | **0** | **1** |
| **0** | **0** | **1** | **0** |
| **1** | **0** | **1** | **0** |
| **0** | **1** | **1** | **0** |
| **1** | **1** | **1** | **0** |

References

1. Shis, D. L., Hussain, F., Meinhardt, S., Swint-Kruse, L. & Bennett, M. R. Modular, multi-input transcriptional logic gating with orthogonal LacI/GalR family chimeras. *ACS Synth Biol* **3**, 645-651 (2014).

2. Stanton, B. C. *et al.* Genomic mining of prokaryotic repressors for orthogonal logic gates. *Nat Chem Biol* **10**, 99-105 (2014).

3. Tamsir, A., Tabor, J. J. & Voigt, C. A. Robust multicellular computing using genetically encoded NOR gates and chemical 'wires'. *Nature* **469**, 212-215 (2011).

4. Hunziker, A., Tuboly, C., Horvath, P., Krishna, S. & Semsey, S. Genetic flexibility of regulatory networks. *Proc Natl Acad Sci U S A* **107**, 12998-13003 (2010).

5. Shin, J. & Noireaux, V. An E. coli cell-free expression toolbox: application to synthetic gene circuits and artificial cells. *ACS Synth Biol* **1**, 29-41 (2012).

6. Karig, D. K., Iyer, S., Simpson, M. L. & Doktycz, M. J. Expression optimization and synthetic gene networks in cell-free systems. *Nucleic Acids Res* **40**, 3763-3774 (2012).

7. Iyer, S., Karig, D. K., Norred, S. E., Simpson, M. L. & Doktycz, M. J. Multi-input regulation and logic with T7 promoters in cells and cell-free systems. *PLoS One* **8**, e78442 (2013).

8. Lewis, M. The lac repressor. *C R Biol* **328**, 521-548 (2005).

9. Semsey, S., Krishna, S., Sneppen, K. & Adhya, S. Signal integration in the galactose network of Escherichia coli. *Mol Microbiol* **65**, 465-476 (2007).

10. Phillips, S. E. *et al.* Cooperative tandem binding of met repressor of Escherichia coli. *Nature* **341**, 711-715 (1989).

11. Liu, Y. C. & Matthews, K. S. Dependence of trp repressor-operator affinity, stoichiometry, and apparent cooperativity on DNA sequence and size. *J Biol Chem* **268**, 23239-23249 (1993).
